# Evaluation of Carboxymethyl Chitosan–Genipin Hydrogels as Reservoir Systems for Suramin Delivery in Epithelial Tissues

**DOI:** 10.3390/gels11050312

**Published:** 2025-04-23

**Authors:** David Encinas-Basurto, Victor H. Ruiz, Rick G. Schnellmann, Heidi M. Mansour

**Affiliations:** 1 Nanotechnology Program, Department of Physics, Universidad de Sonora, Hermosillo 83000, Sonora, Mexico; 2 Skaggs Pharmaceutical Sciences Center, The University of Arizona R. Ken Coit College of Pharmacy, 1703 E Mabel St., Tucson, AZ 85721, USA; 3 Department of Medicine, The University of Arizona College of Medicine, 501 N Campbell Ave., Tucson, AZ 85724, USA; 4 BIO5 Institute, The University of Arizona, 1657 E Helen St., Tucson, AZ 85719, USA; 5 Center for Translational Science, Florida International University, Port St. Lucie, FL 34987, USA

**Keywords:** carboxymethyl chitosan HD, suramin, drug permeation, EpiDerm™ model, controlled release

## Abstract

Hydrogels (HDs) offer a promising platform for localized and sustained drug delivery. In this study, carboxymethyl chitosan (CMC)—based hydrogels were crosslinked with genipin and evaluated for the controlled release and tissue retention of suramin, a polyanionic drug with anti-inflammatory and antifibrotic properties. The influence of crosslinking density (1%, 3%, and 5%) on drug release, permeation kinetics, and retention was investigated using in vitro synthetic membranes and reconstructed human epithelial tissue models. The 1% genipin HD exhibited the highest cumulative release and drug retention (48.8 ± 6.8 μg/cm^2^ in synthetic membranes; 24.06 ± 7.33 μg/cm^2^ in epithelial models), along with a sustained release profile governed by first-order and Fickian diffusion kinetics. Notably, the 1% crosslinked formulation also demonstrated enhanced transmembrane flux (>140 μg/cm^2^/h after six hours), suggesting that lower crosslinking density favors both diffusional mobility and depot functionality. In contrast, free suramin solution displayed limited tissue interaction and minimal permeation, highlighting the role of the hydrogel matrix in regulating local bioavailability. These findings demonstrate that CMC–genipin HD can closely modulate drug delivery kinetics through crosslinking density, offering a biocompatible strategy for localized treatment of ulcerated epithelial conditions such as oral mucositis or chronic wounds. Diffusion models included a synthetic multilayer membrane (Strat-M^®^) and a reconstructed human epidermis (EpiDerm™) to simulate skin-like barrier properties.

## 1. Introduction

Chronic wounds and epithelial ulcers represent a significant clinical challenge due to their prolonged healing process and high susceptibility to infections and inflammation [1,2]. Conditions such as oral mucositis, diabetic foot ulcers, and corneal ulcers require localized and sustained drug delivery strategies to improve treatment efficacy and patient outcomes. Traditional approaches often rely on topical drug applications, which suffer from rapid clearance, low retention, and poor bioavailability, leading to suboptimal therapeutic effects [3]. In this context, hydrogel (HD)—based drug delivery systems have emerged as promising alternatives, offering enhanced drug retention, controlled release, and improved interaction with biological tissues [4].

Among various HDs explored for biomedical applications, chitosan-based HDs have gained significant attention due to their biocompatibility, biodegradability, mucoadhesive properties, and inherent antimicrobial activity. Because of its mucoadhesiveness, biocompatibility, biodegradability, and ability to form HD through chemical or physical crosslinking, which serve as drug reservoirs and allow for controlled diffusion and prolonged retention of therapeutic agents, carboxymethyl chitosan (CMC), a water-soluble, anionic derivative of chitosan, has garnered interest as a drug delivery matrix. Incorporating genipin as a natural crosslinker further enhances the mechanical stability and structural integrity of CMC HDs while maintaining low cytotoxicity, making them ideal candidates for localized drug delivery in wound healing and ulcer management [5]. CMC carboxyl and amino functional groups allow it to interact with various drugs, including negatively charged substances like suramin. Numerous trials have demonstrated CMC hydrogels to preserve drug release, enhance local retention, decrease burst effects, and act as an anti-adhesion solution to prevent postsurgical adhesions, as we have previously published [6,7]. Interestingly, genipin-crosslinked CMC membranes have adjustable mechanical and swelling characteristics, which makes them potential delivery systems for macromolecular and hydrophilic compounds [8].

Suramin, a polysulfonated naphthylurea, has been explored for its therapeutic potential beyond its original use as an antiparasitic drug. Beyond its antiparasitic origin, suramin is pharmacologically notable for its broad-spectrum inhibition of extracellular signaling. It can interfere with various growth factors and cytokines by preventing their binding to respective receptors [9]. This unique mechanism underlies its diverse biological activities, including anti-inflammatory, antifibrotic, and nephroprotective properties, which have been demonstrated in multiple disease models [10]. Studies have shown that suramin mitigates renal injury by modulating cellular pathways associated with fibrosis and inflammation [11,12], protects against cisplatin-induced nephrotoxicity [13], and reduces diabetic nephropathy-related damage [14]. Additionally, suramin has been reported to attenuate ethanol-induced liver injury by reducing oxidative stress, inflammation, and fibrosis, thereby mitigating hepatocellular damage and preserving liver function [15]. Suramin has been demonstrated to inhibit epithelial ulcer development in an experimental type of HSV-1 keratitis, effectively decreasing ulcer size and accelerating re-epithelialization without demonstrating direct antiviral activity [16] and inhibiting excessive scar formation following ocular filtration surgery, suggesting its potential to control fibrosis in specific wound healing contexts [17].

However, its hydrophilicity and rapid systemic clearance limit its clinical application as a free drug solution. HD based on CMC crosslinked with genipin can improve the local delivery of suramin by modulating its release and retention through controlled crosslinking density, thereby enhancing its therapeutic potential for epithelial ulcers. This study contributes to the growing interest in biopolymeric drug delivery systems, particularly CMC-based HDs, which have demonstrated enhanced swelling capacity, mucoadhesion, and tunable drug release profiles. By systematically evaluating the permeation and retention of suramin in model membrane Strat-M^®^ and 3D keratocytes model cell EpiDerm™ models, this work provides key insights into the potential of CMC–genipin HDs for localized ulcer treatment. The comparison of these models enables us to understand better how the HD structure affects drug diffusion across synthetic and biological barriers, highlighting its potential for epithelial wound healing and mucosal drug delivery.

## 2. Results and Discussion

### 2.1. Scanning Electron Microscopy (SEM)

**Figure 1:**
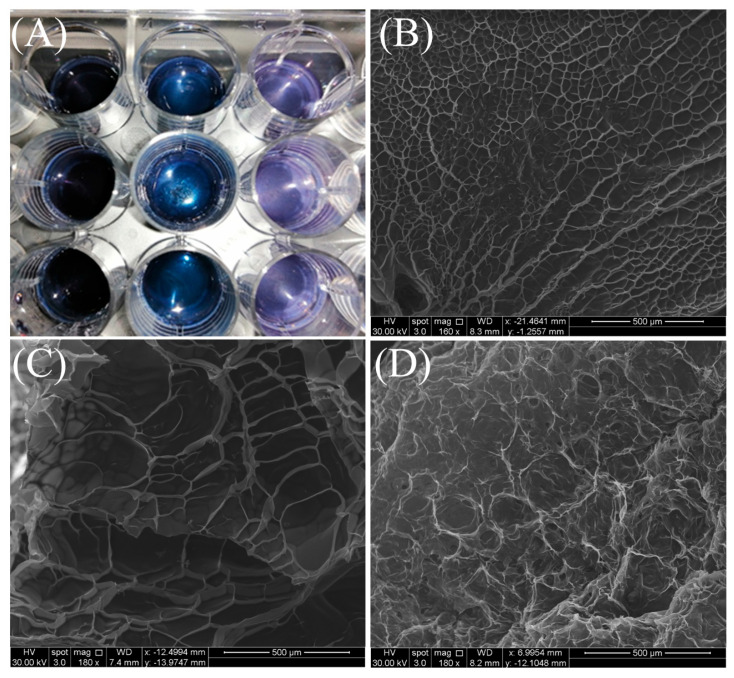
Visual and structural characterization of CMC–genipin HDs. (**A**) Representative images of HDs showing the characteristic dark blue coloration due to genipin crosslinking at concentrations of 1% *w*/*w*, 3% *w*/*w*, and 5% *w*/*w* (right to left). (**B**–**D**) Scanning electron microscopy (SEM) images of freeze-dried HDs at genipin concentrations of 1% (**B**), 3% (**C**), and 5% (**D**) *w*/*w*.

Figure 1A shows that CMC/genipin crosslinking forms a dark-blue HD due to genipin reacting with CMC amino groups. The mechanism has two possible paths. Under moderate acidic or neutral circumstances, crosslinking happens in two steps: a nucleophilic attack of chitosan amino groups on genipin olefinic carbon (C3), followed by ring-opening and amide bond formation. In the second mechanism is proposed that oxygen radicals cause genipin molecules to polymerize, resulting in chitosan chain crosslinking via highly conjugated genipin copolymers, which are responsible for the dark-blue color [18]. HDs crosslinked with varying genipin concentrations (1% *w*/*w*, 3% *w*/*w*, and 5% *w*/*w* relative to CMC) are reviewed in Figure 1. The HDs’ macroscopic appearance on a 12-well plate is shown in Panel A, where a distinct blue coloration gradient is seen. This is probably due to the HDs’ increasing crosslinking as genipin concentrations increase. This shift in color intensity indicates the development of denser polymeric networks. The HD containing 1% genipin’s surface morphology is shown in Panel B. It has a porous structure with large, interconnected channels, which indicates a low crosslinking density. The HD containing 3% genipin is shown by Panel C, which displays a more compact network with smaller and more consistent pores, indicating a moderate crosslinking level. Finally, at 5% genipin, the HD produces a dense framework with minor porosity (Figure 1D), indicating high crosslinking.

Yang, et al. [19] evaluated the synthesis and characterization of CMC HD crosslinked with genipin for controlled 5-Fluorouracil delivery applications. Similarly to our study, the SEM analysis demonstrates that increasing genipin concentration leads to a progressive reduction in pore size and a denser network structure. These morphological changes are connected with functional properties such as reduced swelling and more controlled drug release, making both sets of HDs suitable for applications requiring mechanical stability and sustained release systems. This study highlights the importance of genipin concentration in determining the structural and functional properties of CMC-based HDs. Genipin, a nucleophilic reagent, interacts with the amino groups of CMC to form heterocyclic amino compounds. The amino groups on CMC were primarily changed into heterocyclic amines during genipin crosslinking. Additionally, only primary amines—not secondary or tertiary amines—could react with genipin, and genipin formed blue pigments. pH is the most critical factor influencing blue colorant intensity and reaction kinetics during crosslinking. When genipin spontaneously combines with amino acids and proteins containing primary amines, water-soluble blue pigments are rapidly formed [20].

These findings show that the genipin concentration directly and significantly affects the structural and functional properties of HDs. By varying the crosslinking degree, HDs can be tailored for specific purposes, ranging from high-swelling, flexible systems to dense, brittle materials with improved mechanical stability. This adaptability demonstrates the versatility of genipin-crosslinked CMC HDs as a platform for controlled drug delivery, tissue engineering, and other biomedical applications requiring precise mechanical and diffusion properties.

### 2.2. Swelling Ratio

Table 1 summarizes the swelling ratio (SR) of the HDs crosslinked with different genipin concentrations (1% *w*/*w*, 3% *w*/*w*, and 5% *w*/*w*). The results demonstrate a trend in dramatically reducing the swelling ratio as genipin levels increase. In contrast to HDs crosslinked with 3% and 5% genipin, which show much lower swelling ratios of 63 ± 11% and 41 ± 7%, respectively, HDs crosslinked with 1% genipin show the most significant swelling ratio (205 ± 5%). Because of the higher crosslinking density, the swelling ratio decreases as the genipin concentration rises, suggesting a more substantial and denser network structure.

Del Olmo, et al. [21] valuated the insights into the synthesis and characterization of CS–genipin HDs, focusing on their swelling properties. The authors investigated how synthesis parameters, including reaction time, temperature, and CS–genipin weight ratios, influence the equilibrium swelling ratio. They observed that higher genipin concentrations and prolonged reaction times led to increased crosslinking density, which reduced the swelling capacity of the HDs. Yang, Lan, Guo, Cheng, Fan, Cai, Zhang, Chen and Zhou [19], for instance, reported in a study on CMC-5FU HDs that swelling ratios decreased three-fold with increasing genipin concentrations, highlighting a similar relationship between crosslinking density and water uptake.

The degree of crosslinking, which is controlled by the genipin concentration, has an immediate effect on the HDs’ swelling behavior. The high swelling ratio indicates that the HD network is loosely crosslinked at 1% genipin, creating a more open structure that absorbs and holds a greater volume of water. On the other hand, a denser network with less porosity and less free volume is produced when the genipin concentration is raised to 3% and 5%. This pattern limits water uptake and lowers swelling ratios. These results are consistent with trends reported in other genipin-crosslinked CMC systems, such as those used in ophthalmic drug delivery [19], where increased crosslinker content was associated with decreased swelling and drug release rates.

Controlling the swelling ratio is crucial for applications like tissue engineering, where mechanical stability and hydration are essential, and drug administration, where the swelling behavior affects the release rate of encapsulated drugs, depending on whether high swelling capacity or structural rigidity is prioritized. Applications incorporating controlled release of drugs may benefit from the decreased swelling at higher crosslinking densities due to the limited mobility of the polymer chains and the smaller pore diameters; studies have connected lower swelling ratios to sustained release profiles, which is similar to how HDs behave [22,23].

### 2.3. In Vitro Drug Release

Recent studies have highlighted the efficacy of chitosan-based HDs for the long-term delivery of different drugs in the biomedical area, including overcoming multidrug resistance in cancer cells using nanoparticles, as we have previously published [24,25]. By creating a diffusion barrier, chitosan three-dimensional porous and hydrophilic structure permits the incorporation of drugs while also preventing their fast release [26], the release of integrated drugs by hydrolytic or enzymatic destruction of polymer chains is known as chemically controlled release. Diffusion-controlled release happens when the HD swelling exceeds the diffusion rate, whereas swelling-controlled release happens when molecular diffusion is slower than the HD swelling [27,28,29].

**Figure 2:**
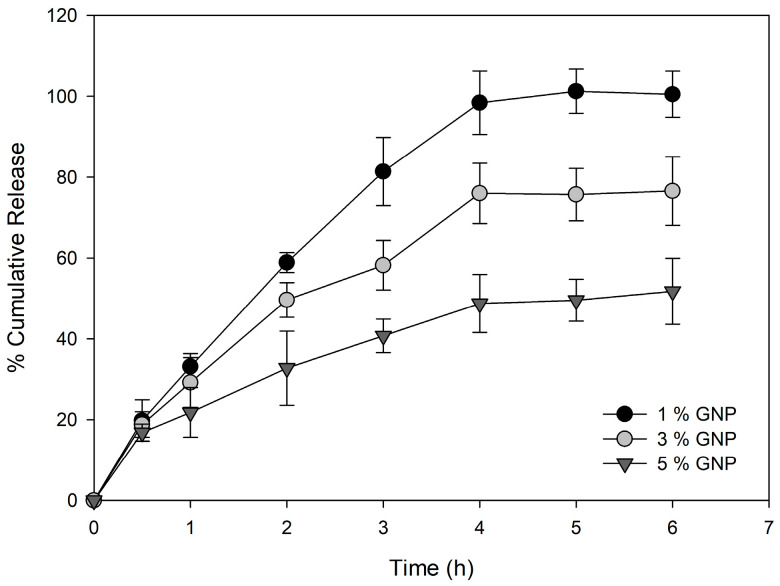
Cumulative release profiles of suramin from CMC–genipin HDs at different crosslinking densities (1%, 3%, and 5% genipin *w*/*w*).

The cumulative release profiles of suramin from genipin-crosslinked HDs reveal a clear dependence on the crosslinking degree (Figure 2). HDs with 1% genipin showed the fastest and most complete release (98% in 4 h), attributed to their low crosslinking density and highly porous network, allowing rapid diffusion. HDs with 3% genipin had a moderate release (80% in 4 h), whereas those with 5% genipin had the slowest and most sustained release (40% in 4 h) because of their dense, compact shape, which hinders drug diffusion. These findings are consistent with the swelling ratio trends, indicating that more significant genipin concentrations reduce HD porosity and water uptake. The tunability of these HDs makes them suitable for varied applications, with low genipin concentrations ideal for rapid drug release and higher concentrations for sustained delivery systems, depending on therapeutic needs. The cumulative suramin release at 6 h was statistically examined for the 1%, 3%, and 5% genipin CMC hydrogel formulations to assess the impact of genipin crosslinking on drug release in more detail. A significant difference between the three groups was found using one-way ANOVA (*p* < 0.05). The Tukey’s test post hoc analysis showed significant differences between all treatments (*p* < 0.05).

Drug diffusion is regulated by the pore size of the HD matrix; the higher the swelling rate, the larger the pores, and the more rapidly the small-sized drugs diffuse through the HD. On the other hand, the release of macromolecules is usually delayed due to their larger hydrodynamic radius [30,31,32]. Emani, et al. [33] studied HDs based on CMC crosslinked covalently with trimesic acid to control the release of a hydrophilic drug, likely 5-fluorouracil (5-FU); similar to our observations, different trimesic acid concentrations were used to tune the degree of crosslinking, allowing for systematic control of the HD properties. Dissolution studies conducted in this work were performed under simulated physiological conditions (PBS, pH 7.4, at 37 °C) to mimic the in vivo environment and assess the release behavior of suramin from the hydrogels. HDs with higher crosslinker concentrations exhibited slower release profiles due to the denser network, and these findings support the hypothesis that lower crosslinking densities enhance drug diffusion due to a looser hydrogel network. In contrast, higher genipin content leads to more compact matrices restricting drug mobility. The clear statistical separation between the three formulations emphasizes the tunability of drug release kinetics through crosslinker concentration. Although the release study was conducted over 6 h, the data suggest that some HD formulations, particularly those with higher crosslinking densities, were still in the active phase of drug release at the final time point (79% for 5% *w*/*w* GNP CMC HDs).

Although the cumulative release profile of the 3% *w*/*w* genipin-crosslinked HD appears to plateau after 4 h, a modest rising tendency remains visible in the latter time points. This behavior is consistent with strongly crosslinked hydrogel systems, where the denser polymeric network limits water intake and molecule diffusion. In these settings, the release is predominantly driven by Fickian diffusion, and residual suramin is likely trapped within the inner matrix or held by ionic interactions with the cationic chitosan chain [34]. Similar biphasic release behavior has been seen with genipin-crosslinked chitosan matrices containing polyanionic drugs, with a quick burst release followed by an extended gradual release phase extending more than 20 h [35].

### 2.4. Drug Release Kinetics

**Figure 3:**
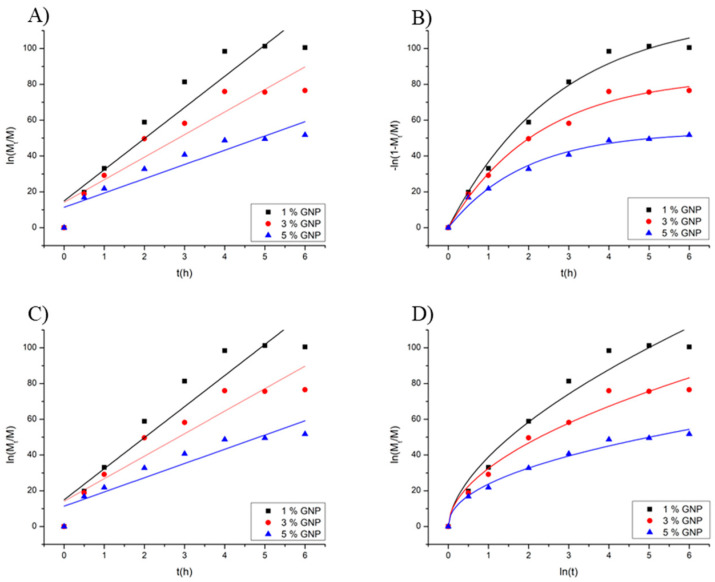
Fitting of suramin release data to different kinetic models: (**A**) zero-order, (**B**) first-order, (**C**) Higuchi, and (**D**) Korsmeyer–Peppas, for CMC–GNP hydrogels loaded with suramin at 1%, 3%, and 5% GNP.

The suramin release profiles from genipin-crosslinked CMC HDs showed distinct routes according to the crosslinking degree (Table 2). The first-order model, which best accounted for the data for HDs with 1% and 3% genipin (R^2^ > 0.98), showed that the release rate was primarily proportional to the remaining drug concentration and diffusion-controlled. This behavior is consistent with lower crosslinked HDs’ porous character, where water penetration promotes drug diffusion [36]. The first-order and Korsmeyer–Peppas models fit the release data well for the 5% genipin HD (R^2^ = 0.974 and 0.978, respectively). The Peppas model produced a diffusion exponent (n = 0.46), indicating a Fickian diffusion process despite the slight difference in correlation coefficients. This is in accordance with the formulation’s small swelling behavior, which is primarily controlled by concentration gradients due to the high crosslinking density, which restricts matrix relaxation. As a result, the Peppas model supports the physical interpretation of the release mechanism in these circumstances and provides a good fit to the data (Figure 3).

Del Olmo, Pérez-Álvarez, Sáez-Martínez, Benito-Cid, Ruiz-Rubio, Pérez-González, Vilas-Vilela and Alonso [21] studied the development of CMC HD crosslinked with polyethylene glycol succinimidyl carbonate. Considering that the Korsmeyer–Peppas and Higuchi models dominate, they demonstrate how crosslinking density plays a crucial role in controlling the mechanisms of vitamin C release, with highly crosslinked HDs showing slower, diffusion-controlled release. Our findings agree with the reported change from anomalous diffusion at lower crosslinking levels to Fickian diffusion at higher crosslinking levels, highlighting the adaptability of CMC HDs in regulating hydrophilic drug release rates. These similarities suggest these HDs’ suitability for controlled drug delivery systems where exact release profile modification is essential.

### 2.5. In Vitro Cell Viability of 2D Cell Culture of a Human Skin Cell Line and Human Primary Skin Cells

**Figure 4:**
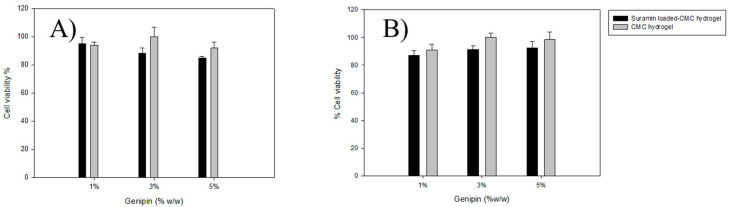
Cell viability of HaCaT (**A**) and TR146 (**B**) cells after 24 h of exposure to suramin-loaded and unloaded CMC–genipin hydrogels at different genipin concentrations (1%, 3%, and 5% *w*/*w*). Data represent mean ± SD (n = 3). Cell viability was evaluated using the MTT assay.

In this study, the CMC HDs were crosslinked with genipin to generate a biocompatible substrate for suramin distribution that is targeted and maintained. The HDs showed significant cell vitality in both HaCaT keratinocytes (oral mucosal model) and TR-146 epithelial cells (buccal mucosal model), with viability surpassing 85% for all formulations, including suramin-loaded HDs (Figure 4). For HaCaT cells, both loaded and unloaded HDs showed high cell viability, ranging from 90% to 100% across all crosslinking densities. Higher genipin concentrations resulted in a modest reduction in cell viability, but the HDs remained highly biocompatible. Similarly, TR-146 cells showed comparable trends, with cell viability above 85% for all formulations. While the suramin-loaded HDs exhibited marginally lower viability than unloaded HDs, the overall results confirm the non-cytotoxic nature of the HDs and their suitability for biomedical applications.

Additionally, the HDs’ crosslinking density modulates suramin release, with higher genipin concentrations (5% *w*/*w*) yielding a slower, diffusion-controlled release suitable for chronic conditions requiring prolonged therapeutic action. Conversely, lower crosslinking densities (1% and 3% *w*/*w*) facilitate faster release, potentially addressing acute inflammatory phases in oral mucositis or early-stage wound management in foot ulcers. de Lacerda Bukzem, Dos Santos, Leite, Inada and Campana-Filho [8] evaluated the biocompatibility of CMC and PVA membranes crosslinked with genipin using human dermal fibroblasts (HDF) and keratinocytes (HaCaT) as cell models. Cell viability was evaluated to determine that the membranes did not have cytotoxic properties. The results revealed excellent cell viability in all tested formulations, indicating their compatibility with human cells. Genipin is commonly used in biomedical applications because it avoids the toxicity associated with synthetic crosslinkers such as glutaraldehyde. The combination of biocompatibility, sustained drug release, and the ability to tailor delivery profiles underscores the potential of suramin-loaded CMC HDs as a versatile therapeutic strategy. These HDs help reduce inflammation, promote tissue healing, and improve patient outcomes in oral mucositis and foot ulcer care because they maintain localized therapeutic levels while decreasing systemic exposure.

Chitosan has been demonstrated to be a highly effective polymer for drug delivery in buccal tissue due to its mucoadhesive, biocompatibility, and permeation-promoting properties [37,38]. Its cationic nature allows for considerable interactions with the negatively charged mucin in epithelial tissue, extending retention time and improving drug absorption by avoiding enzymatic degradation and first-pass metabolism. Furthermore, chitosan briefly opens tight connections in the epithelium, allowing macromolecules to be delivered and improving the effective delivery of hydrophilic drugs and peptides [39]. Sander, et al. [40] investigated the potential of metformin delivery via the buccal mucosa using a TR146 cell culture model. The researchers tested bioadhesive chitosan disks and chitosan solutions to enhance the permeability of metformin, a hydrophilic drug, across the buccal epithelium. The bioadhesive tablets were tested for cytotoxicity on TR146 cells, showing good compatibility at lower chitosan concentrations. However, higher concentrations of chitosan in the formulation reduced cell viability and disrupted epithelial integrity, highlighting the need to optimize polymer content for safety.

### 2.6. In Vitro Permeation of Suramin Using Strat-M^®^ Synthetic Biomimetic Membrane and Franz Cell Diffusion System

**Figure 5:**
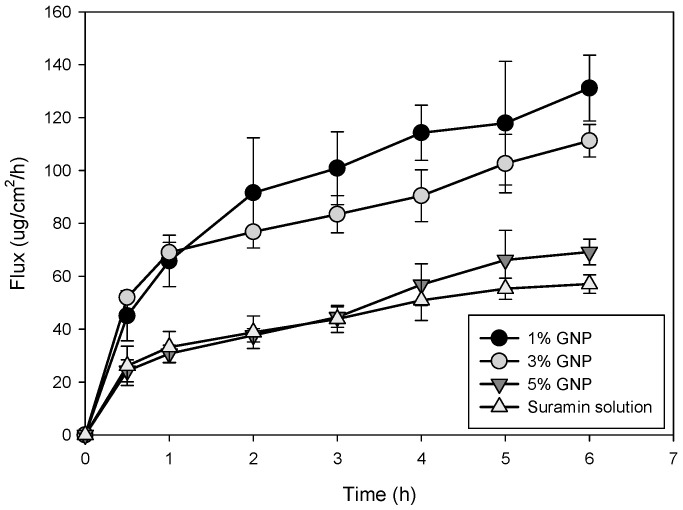
Flux profiles of suramin across Strat-M^®^ membranes from suramin solution and CMC–genipin HDs with varying crosslinking densities (1%, 3%, and 5% genipin *w*/*w*).

Franz diffusion cells containing CMC HDs crosslinked with 1%, 3%, and 5% genipin were used to assess the suramin permeation flux across the Strat-M^®^ membrane over 6 h (Figure 5). A plain suramin solution was used as a comparison. The permeation barrier was the synthetic multilayered membrane known as Strat-M^®^, created to mimic human skin characteristics. Its structure provides a reliable and consistent model for drug absorption studies by simulating the moist dermis and the lipid-dense stratum corneum [41].

After six hours, the HD with 1% genipin crosslinking had the most significant flux, exceeding 140 μg/cm^2^/h. The 5% crosslinked HD had the lowest flux of the HD formulations, followed by the 3% crosslinked HD, which displayed a moderate flux. The flux of the plain suramin solution was significantly lower than that of other HD systems, highlighting the role that CMC HDs play in enhancing suramin penetration. Increased drug release was seen at lower crosslinking densities (1% and 3%), most likely due to more porous network architectures that made it easier for suramin to diffuse. In contrast, the 5% crosslinked HD had a denser matrix, which limited drug diffusion and resulted in a slower, prolonged release profile. Using the Strat-M^®^ membrane allowed for accurate and reproducible penetration measurement, revealing CMC HDs’ potential to regulate drug delivery through controlled crosslinking density changes.

Table 3 presents flux and drug retention results, illustrating the influence of genipin crosslinking density on suramin delivery across the Strat-M^®^ membrane. The 1% (*w*/*w*) genipin HD showed the highest flux (12.69 ± 4.34 μg/cm^2^/h) and drug retention (48.8 ± 6.8 μg/cm^2^), indicating that its porous structure facilitated rapid diffusion and membrane contact. While the 5% (*w*/*w*) genipin HD decreased drug diffusion because of its denser matrix, resulting in the lowest retention (5.75 ± 0.4 μg/cm^2^), the 3% (*w*/*w*) genipin HD demonstrated moderate retention (31.61 ± 0.18 μg/cm^2^) and flux (8.09 ± 2.61 μg/cm^2^/h). Interestingly, the suramin solution’s flux and drug retention values were statistically comparable to those of the 5% HD (*p* > 0.05), even though it had no retention impact from the hydrogel matrix. This implies that drug release and membrane contact may be limited to values similar to those of the free drug in solution by high crosslinking density. The 1% HD, however, showed the potential for faster delivery because it varied significantly from all other formulations in flux and drug retention (*p* < 0.05).

These results coincide with the principles of controlled drug delivery, wherein HD serves as reservoirs that regulate drug availability and maintain release [42]. Drug permeation could be precisely and consistently measured using the Strat-M^®^ membrane, demonstrating the ability of genipin-crosslinked CMC HDs to control crosslinking and modify drug delivery. The one-way ANOVA and Tukey’s post hoc analysis further supported this, confirming that the 1% HD’s flux values were significantly higher than those of the 3%, 5%, and 0% groups (*p* < 0.05). Despite having different processes, there was no statistically significant difference in flux between the suramin solution and the 5% HD (*p* > 0.05), suggesting similar permeation performance.

The free suramin solution’s flux and retention data (Table 3) offer crucial information on the drawbacks of unformulated or free-drug delivery. Despite its rapid diffusion over the membrane (flux: 6.02 ± 2.68 μg/cm^2^/h) and free solubility, suramin retention is much lower than that of the hydrogel formulations. Given that free suramin is hydrophilic and polyanionic, which restricts partitioning or binding inside the membrane’s lipid domains, it appears that although it permeates the membrane effectively, it interacts and associates with the membrane surface considerably [43]. The CMC–genipin hydrogels, on the other hand, provide a controlled release reservoir that improves retention while maintaining drug diffusion. Prolonged contact times and localized drug accumulation at the membrane interface are probably the causes of this higher retention. According to previous studies, HD-based delivery slows drug release and increases local bioavailability, which is not possible with free suramin because of its poor tissue contact and clearance [44]. In addition to highlighting the clinical significance of retention as a crucial parameter in drug delivery design, this contrast underscores the HD system’s functional benefit in facilitating localized, prolonged drug action.

These results show the physiological processes controlling drug diffusion and retention in hydrophilic and charged environments. A highly hydrophilic molecule with several sulfonate groups, suramin matches the behavior of mucosal tissues by efficiently interacting with the hydrophilic portions of the membrane [45]. The behavior demonstrates how genipin-crosslinked HDs can be used to create customized drug administration patterns that might be modified for clinical use to satisfy specific therapeutic requirements; larger crosslinking densities would allow for the more prolonged release of the drug, while lower densities would allow for faster drug delivery. Future research might examine these devices’ biocompatibility in vivo and assess how well they function in dynamic settings like the buccal cavity or wound sites further to confirm their potential in the targeted administration of drugs. Consequently, the HDs provided a controlled release mechanism, ensuring prolonged membrane retention while minimizing rapid drug depletion. These findings align with previous studies demonstrating the role of HD networks in sustaining drug delivery at biological barriers, effectively modulating permeation and retention profiles [46].

### 2.7. In Vitro Permeation of Suramin Using 3D Normal Human-Derived Epidermal Keratinocytes (EpiDerm™)

**Figure 6:**
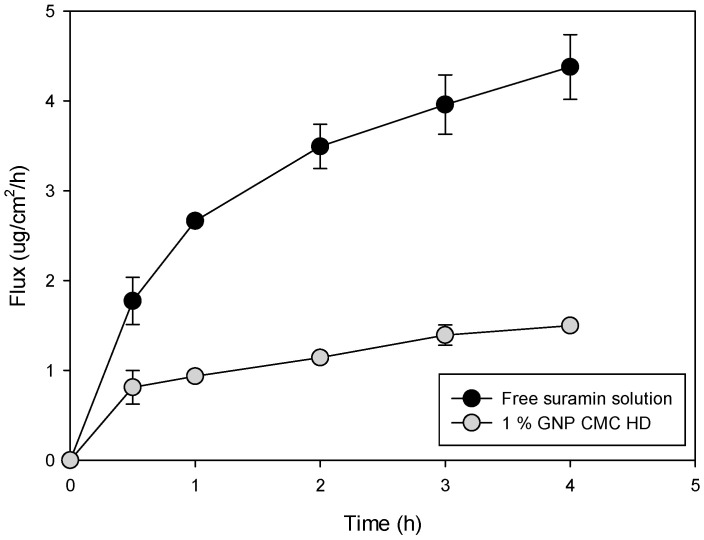
Flux profiles of suramin through EpiDerm™ 3D tissue models from free suramin solution and 1% genipin CMC HD.

The permeation of suramin through a 3D epithelial cell model (EpiDerm™) was evaluated using a free suramin solution and a 1% genipin-crosslinked CMC HD (1% genipin CMC HD). The flux data show that the free suramin solution achieved a significantly higher permeation rate compared to the HD formulation (*p* < 0.05) (Figure 6). Over a 4 h period, the free suramin solution showed a continuous increase in flux, reaching roughly 4.5 µg/cm^2^/h, while the 1% genipin HD showed a much lower and more controlled flux, peaking at around 1.2 µg/cm^2^/h. This difference emphasizes the role of the HD matrix in restricting and controlling suramin diffusion.

The flux and drug retention values obtained from the EpiDerm™ model and the Strat-M^®^ membrane experiments highlight significant differences in the behavior of suramin when delivered through HDs versus free drug solutions. In the EpiDerm™ model, the flux for the 1% genipin HD was significantly lower than that of the free solution (*p* < 0.05), stabilizing at approximately 1.2 μg/cm^2^/h. In contrast, the free suramin solution reached a much higher flux of 4.5 μg/cm^2^/h. These differences highlight the more restrictive diffusion observed in the EpiDerm™ model due to its biological complexity, while the Strat-M^®^ membrane offers a simplified diffusion pathway suitable for initial permeation studies.

The permeation and retention data obtained from the Strat-M^®^ membrane and EpiDerm™ models highlight the significant advantages of CMC–genipin HDs for localized drug delivery compared to free suramin solutions. In the EpiDerm™ model, the flux values for the free suramin solution (0.42 ± 0.15 μg/cm^2^/h) and the HD formulation (0.37 ± 0.04 μg/cm^2^/h) were relatively similar. The delivery method of suramin plays a crucial role in its therapeutic effectiveness against ulcers. While free suramin solutions can rapidly diffuse from the ulcer site, leading to lower localized retention. HD formulations provide a sustained release, enhancing drug bioavailability and prolonged interaction with damaged epithelial tissue. This is evidenced by our EpiDerm™ permeation study, where suramin-loaded HDs retained 24.06 ± 7.33 µg/cm^2^, significantly more than the free suramin solution (13.04 ± 4.33 µg/cm^2^, *p* < 0.05). The HD matrix ensures gradual diffusion, preventing the burst release effect, which could otherwise lead to cytotoxicity or rapid clearance from the ulcer surface.

In contrast, the Strat-M^®^ membrane experiments revealed higher flux values for the free solution and the HD. For the 1% genipin HD, the flux was 12.69 ± 4.34 μg/cm^2^/h, and for the free suramin solution, it was 6.02 ± 2.68 μg/cm^2^/h. The retention values were also greater, with the 1% genipin HD retaining 48.8 ± 6.8 μg/cm^2^ compared to 15.56 ± 3.99 μg/cm^2^ in the free solution. These differences between the models underscore the higher complexity of the EpiDerm™ tissue barrier, which better mimics in vivo conditions and presents a more restrictive environment for drug permeation and retention. The enhanced retention observed with the HD in both models emphasizes its role as a reservoir system, allowing for gradual suramin or other hydrophilic drug release and sustained interaction with the tissue [47].

Additionally, CMC-crosslinked genipin HDs exhibit significant bioadhesive and protective characteristics, provided that suramin is in direct contact with the ulcerated epithelium for an extended period. These characteristics enhance the potential of HD-based formulations in treating chronic ulcers by mimicking the physiologically necessary moist environment for the most effective possible wound healing. This behavior has physiological benefits, especially when treating localized disorders such as oral mucositis or epithelial ulcers, where treatment success depends on drug availability [48]. Additionally, HD minimizes systemic absorption, reducing the potential for adverse effects associated with free suramin solutions.

The Strat-M^®^ and Epi-Derm™ models’ different barrier characteristics are responsible for the variations in flux and drug retention observed between both. With its lipid-rich structure and many layers, Strat-M^®^ is a synthetic membrane replicating the stratum corneum and providing a simplified yet repeatable model of skin permeability. On the other hand, the stratified human keratinocytes that makeup EpiDerm™ form viable cell layers and tight connections that more closely resemble in vivo epithelial barriers. The reduced flux observed with EpiDerm™, which reflects a more constrictive and physiologically appropriate environment for drug penetration, is probably caused by this structural complexity. While EpiDerm™, which more closely approaches human tissue barriers, demonstrated more restricted suramin diffusion and higher retention, especially with hydrogel formulations, Strat-M^®^ synthetic, lipid-based nature permitted higher flux. These results support the validity of applying both models in a complementary manner for drug permeation research.

## 3. Conclusions

This study demonstrates that CMC–genipin HDs effectively modulate the permeation and retention of suramin across biological barriers, providing a controlled and sustained drug release system. The results obtained from Strat-M^®^ and EpiDerm™ models confirm that HD formulations significantly enhance localized drug retention compared to free suramin solutions, with 1% genipin-crosslinked HDs achieving the highest retention (24.06 ± 7.33 µg/cm^2^ in EpiDerm™ and 48.8 ± 6.8 µg/cm^2^ in Strat-M^®^). The lower flux observed in EpiDerm™ (0.37 ± 0.04 µg/cm^2^/h) highlights the role of the epithelial barrier in limiting drug diffusion, emphasizing the importance of HD-based delivery systems in prolonging drug interaction with target tissues. The capacity of these HDs to act as drug reservoirs guarantees that suramin is available at the application site, which is crucial for ulcer treatment and mucosal tissue healing. The HD system is a viable alternative for localized therapy of epithelial ulcers, oral mucositis, and chronic wounds because it reduces systemic absorption while limiting the risk of burst release.

## 4. Materials and Methods

### 4.1. Production of CMC HD

Carboxymethyl chitosan (Santa Cruz Biotechnology Inc. Santa Cruz, CA, USA, Fischer Scientific, Deacetylation degree 90%, Hampton, NH, USA) was prepared at a concentration of 2% (*w*/*v*) by dissolving the polymer in deionized water under constant stirring, as previously reported, with some modifications [49]. Genipin (Sigma Aldrich, St. Louis, MO, USA) was added dropwise to the CMC solution under continuous stirring to ensure homogeneous distribution at 1%, 3%, and 5% (*w*/*w* of CMC) concentration to induce crosslinking. Additionally, 100 µL of suramin solution (20 mg/mL, Sigma Aldrich, USA) was incorporated into each formulation. The mixtures were stirred thoroughly to ensure homogeneity for 5 min, then poured into sterile 12-well plates for shaping, and the subsequent crosslinking reaction was allowed to proceed at room temperature overnight, resulting in CMC HD with varying degrees of crosslinking. The obtained CMC hydrogels were dried at 37 °C for 48 h under ambient conditions until constant weight was achieved, allowing for structural stabilization and solvent removal without thermal degradation of the polymer network and further characterization. The resulting hydrogels exhibited a bulk gel geometry in soft disks shaped by the dimensions of the 12-well plates used for casting.

### 4.2. Scanning Electron Microscopy (SEM)

SEM (FEI, Brno, Czech Republic) was used to examine the HDs’ surface morphology. To apply a 7 nm thin layer of gold–palladium alloy under argon plasma using an Anatech Hummer 6.2 sputtering machine (Union City, CA, USA), samples were mounted on stubs using double-sided sticky carbon tape (Ted Pella, Inc., Redding, CA, USA). SEM pictures were taken at a working distance of roughly 9–12 mm and an accelerating voltage of 30 kV. Digitally, SigmaScan™ Pro 5.0.0 (Systat, Inc., San Jose, CA, USA) was used to calculate the observed HD structures’ mean size, standard deviation, and size range.

### 4.3. CMC HD Swelling Ratio

The swelling ratio (SR) of crosslinked CMC HDs was measured by immersing the samples in 0.1X PBS, pH 7.4 (Sigma-Aldrich, St. Louis, MO, USA) at 37 °C to replicate physiological circumstances. HD samples were dried, cut into little squares (10 mm × 10 mm), weighed (dry weight, *Wd*), and submerged in PBS. The swelled HDs were carefully removed at certain intervals, gently pressed between two paper filters to remove excess buffer, and instantly weighed (*Ws*). The swelling ratio (*SR*) was determined using the following equation:

(1) $$SR=\frac{Ws-Wd}{Wd}$$

The experiment was conducted in triplicate for each HD formulation containing different genipin crosslinking concentrations (1%, 3%, and 5% *w*/*w* of CMC) to evaluate the effect of crosslinking density on the swelling behavior.

### 4.4. In Vitro Suramin Release

The release experiment from HDs was performed using a dialysis membrane (molecular weight cutoff ~3500, Spectrum Laboratories, Inc., Rancho Dominguez, CA, USA) placed in Franz diffusion cells with a diffusion area of 0.64 cm^2^ and a receptor volume of 5 mL. The receptor chamber contained PBS, maintained at 37 °C, and uniform mixing was achieved through magnetic stirring. A 1 mL sample was withdrawn from the receptor chamber at appropriate intervals and immediately replaced with an equal volume of fresh distilled water to maintain sink conditions. The withdrawn samples were analyzed to quantify suramin release over time. The suramin concentration in the collected samples was determined using the HPLC method described in Section 4.6. According to the following equation, the cumulative amount of drug released was determined by adjusting for sample replacement at each time point:

(2) $$M_{t}=C_{n}V+\overset{n-1}{\underset{i-1}{\sum }}C_{i}V_{s}$$

where *M*t** is the cumulative amount of drug released at time t; *C_n_* is the suramin concentration in the receptor medium at the current time point; *C_i_* is the concentration in each previous sample; *V* is the receptor volume (5 mL); and Vₛ is the sample volume (1 mL). The data were reported as a percentage of cumulative release relative to the initial suramin in the hydrogel.

### 4.5. Kinetic Analysis of Suramin Release

The release mechanisms were identified by fitting the release data to zero-order, first-order, Higuchi, and Korsmeyer–Peppas models. The kinetic parameters—including the diffusion exponent (n) and rate constants (k)—were determined using curve fitting. OriginLab Corporation’s OriginPro 2023 was used to undertake kinetic modeling of suramin release profiles in Northampton, Massachusetts, USA. It was determined that the model that best described the release mechanism had the highest R^2^ value.

The release profiles of suramin from CMC hydrogels were analyzed to elucidate the underlying release mechanisms:

Zero-order model:

(3) $$Q_{t}=Q_{0\;}+k_{0}t$$

This model describes a constant release rate, where *Q_t_* is the cumulative amount of drug released at time *t*; *Q*_0_ is the initial drug amount; and *k*_0_ is the zero-order release constant.

First-order model:

(4) $$lnQ_{t}=lnQ_{0}-k_{1}$$

This equation characterizes release dependent on drug concentration, where *k*_1_ is the first-order release rate constant.

Higuchi model:

(5) $$Q_{t}=K_{H}\sqrt{t}$$

This model describes diffusion-controlled release from an insoluble matrix, with *k_H_* representing the Higuchi constant.

Korsmeyer–Peppas model:

(6) $$\frac{Q_{t}}{Q_{\infty }}=k_{p}t^{n}$$

This semi-empirical model identifies the release mechanism based on the diffusional exponent *n*, where *Q*_∞_ is the total drug released at equilibrium; *k_p_* is the kinetic constant; and n determines the release type (Fickian diffusion, non-Fickian transport).

### 4.6. High-Performance Liquid Chromatography (HPLC) Analysis

An Apollo C18 column(150 × 4.6 mm, 5 µm) and a UV–Vis dual wavelength detector were used in conjunction with a Shimadzu LC-2010A HT liquid chromatograph (Torrance, CA, USA) to quantify suramin using a proven HPLC method [50]. Klecker Jr and Collins, 1985. The mobile phase comprised the 5 mM tetrabutyl ammonium phosphate monobasic (TBAP) and 10 mM ammonium acetate buffer in methanol; 30% methanol was present in solution A, while 90% methanol was present in solution B. The gradient program started with 20% solution B, linearly increasing to 70% over 15 min, held at 70% for 5 min, and returned to 20% by minute 20 at a 1 mL/min flow rate. Suramin was dissolved in water for calibration curve preparation (0.1–0.001 mg/mL), and quantification was based on peak area using UV detection at 313 nm.

### 4.7. In Vitro Cell Viability of Human Primary Skin Cells and a Human Skin Cell Line Cultured in 2D

As 2D cell culture models, the cytotoxicity of the HDs was assessed using HaCaT human keratinocyte immortalized cells (AddexBio, San Diego, CA, USA) and TR-146 cells (derived from a 67-year-old female’s neck node, Sigma-Aldrich). HaCaT cells were cultivated in Dulbecco’s Modified Eagle’s Medium (DMEM, Optimized 1X), which was enhanced with Pen-Strep (100 units/mL streptomycin, 100 µg/mL penicillin) and 10% (*v*/*v*) fetal bovine serum (FBS). Pen-Strep and 10% (*v*/*v*) FBS were added to RPMI-1640 medium to sustain TR-146 cells. The two cell lines were incubated at 37 °C with 5% CO_2_ in a humidified environment. In a 6-well plate, 100,000 HaCaT and TR-146 cells were seeded per well. After 24 h, 100 µL of HD and 100 µL of suramin solution (10 mg/mL) were added to the treatment wells. Following a 48 h exposure period and HD removal, each well received 20 µL of a 20 µM resazurin sodium salt solution, which was then incubated for four hours. Using a Molecular Devices^®^ SpectraMax^®^ M3 Multi-Mode Microplate Reader (Sunnyvale, CA, USA), the fluorescence intensity of resorufin was measured at 544 nm (excitation) and 590 nm (emission). The following formula was used to calculate the relative cell viability:

(7) $$RelativeViability\%=\frac{Sample\;flourescence\;intensity}{Control\;flourescece\;intensity}\times 100\%$$

### 4.8. In Vitro Permeation of Suramin Using Strat-M^®^ Synthetic Biomimetic Membrane and Franz Cell Diffusion System

The permeation and retention of suramin through a Strat-M^®^ synthetic membrane (Sigma Aldrich, St. Louis, MO, USA) were evaluated using a glass Franz Diffusion Cell system (PermeGear, Hellertown, PA, USA). The experiment was conducted with suramin formulations prepared as described in the Section 4. The diffusion area of the membrane was 0.64 cm^2^, and the receptor compartment was filled with 5 mL of phosphate-buffered saline (PBS, pH 7.4) as the receptor medium. The receptor medium was maintained at 32.0 ± 0.5 °C, representing the physiological skin temperature, using a reciprocal shaking bath model 25 (Thermo Fisher Scientific, Fair Lawn, NJ, USA) at 30 oscillations per minute. A 200 µL of suramin solution (1% *w*/*v*) was applied onto the Strat-M^®^ membrane. At predetermined intervals, 200 µL of receptor medium was sampled and immediately replaced with an equal volume of fresh medium to maintain sink conditions. The linear regression slope of the linear part of the permeation curve was used to calculate the flux at steady state (J). The HPLC method outlined in the Section 4 examined the cumulative drug penetration and retention on the membrane. To ensure reproducibility, every experiment was carried out three times. The Strat-M^®^ membrane from the Franz cell was submerged in PBS, sonicated for 10 min with a probe sonicator, and then centrifuged at 1400 rpm and 25 °C. A quantitative HPLC analysis was carried out following supernatant filtration.

### 4.9. Utilizing 3D Normal Human-Derived Epidermal Keratinocytes (EpiDerm™) for Suramin Permeation In Vitro

MatTek’s methodology assessed the suramin’s penetration into the EpiDerm™ tissue model using the MatTek Permeation Device (MatTek, Ashland, MA, USA). One milliliter of Dulbecco’s PBS without calcium chloride (CaCl_2_) or magnesium chloride (MgCl_2_) was added to each well of a 6-well cell culture plate before the EpiDerm™ samples were deposited onto it in tissue culture inserts (MatTek, Ashland, MA, USA). In a reciprocal shaking bath model 25 (Thermo Fisher Scientific, Fair Lawn, NJ, USA) with 30 oscillations per minute, the plate was kept at 37 °C (physiological temperature). A volume of 400 µL of suramin formulation (e.g., HD or solution) was applied to the surface of the EpiDerm™ tissue. A diffusion area of 0.256 cm^2^ was effective. The sink conditions were maintained by removing 200 µL samples from the receiver medium at predefined intervals and replacing them with an equivalent volume of fresh PBS. The cumulative penetration of suramin was measured using the previously mentioned HPLC approach. Plotting the cumulative amount of suramin-permeated 3D model cells versus time allowed for the determination of the steady-state flux (J), which was the slope of the linear part of the plot.

### 4.10. Statistical Analysis

Experimental data are presented as mean ± standard deviation (SD) from triplicate measurements (n = 3). One-way analysis of variance (ANOVA) was performed to suramin flux across membranes and drug retention. When statistically significant differences were detected (*p* < 0.05), Tukey’s post hoc test was applied to identify pairwise differences between formulations. Statistical computations were carried out using SPSS version 27 (IBM).

## Figures and Tables

**Table 1:** The swelling ratio of CMC–genipin HDs at different crosslinking densities.

Genipin % *w*/*w*	Swelling Ratio (%)
1	205 ± 5.1
3	63 ± 11.6
5	41 ± 7.2

**Table 2:** Kinetic modeling parameters for suramin release from CMC–genipin HDs at different crosslinking densities.

Sample % Genipin	Higuchi k	Higuchi R^2^	k0	R^2^	First-Order k1	R^2^	Peppas k	n	R^2^
1%	43.75	0.93	15.57	0.89	0.36	0.98	38.88	0.58	0.94
3%	33.59	0.95	10.88	0.89	0.44	0.98	32.47	0.52	0.95
5%	22.58	0.97	6.58	0.92	0.52	0.97	23.72	0.46	0.98

**Table 3:** Flux and drug retention values for suramin across Strat-M^®^ membranes. Different superscript letters indicate statistically significant differences (*p* < 0.05) according to Tukey’s post hoc test.

Sample (% Genipin)	Flux (µg/cm^2^/h)	Drug Retention in Membrane (µg/cm^2^)
1%	12.7 ± 4.3 ^a^	48.8 ± 6.8 ^a^
3%	8.0 ± 2.6 ^b^	31.61 ± 0.1 ^b^
5%	6.5 ± 1.3 ^b,c^	5.75 ± 0.4 ^c^
Suramin Sol	6.0 ± 2.6 ^c^	15.5 ± 3.9 ^c^

## Data Availability

The original contributions presented in the study are included in the article; further inquiries can be directed to the corresponding author.

## Abbreviations

The following abbreviations are used in this manuscript:

CMC	Carboxymethyl chitosan
SR	Swelling ratio
HDF	Human dermal fibroblasts
SEM	Scanning electron microscopy
Wd	Weighed
Ws	Immediately weighed

## References

[1] Raffetto J.D., Ligi D., Maniscalco R., Khalil R.A., Mannello F. (2020). Why venous leg ulcers have difficulty healing: Overview on pathophysiology, clinical consequences, and treatment. J. Clin. Med..

[2] Zhao R., Liang H., Clarke E., Jackson C., Xue M. (2016). Inflammation in chronic wounds. Int. J. Mol. Sci..

[3] Samiraninezhad N., Asadi K., Rezazadeh H., Gholami A. (2023). Using chitosan, hyaluronic acid, alginate, and gelatin-based smart biological hydrogels for drug delivery in oral mucosal lesions: A review. Int. J. Biol. Macromol..

[4] Kesharwani P., Bisht A., Alexander A., Dave V., Sharma S. (2021). Biomedical applications of hydrogels in drug delivery system: An update. J. Drug Deliv. Sci. Technol..

[5] Mndlovu H., du Toit L.C., Kumar P., Choonara Y.E., Marimuthu T., Kondiah P.P., Pillay V. (2020). Bioplatform fabrication approaches affecting chitosan-based interpolymer complex properties and performance as wound dressings. Molecules.

[6] Fiamingo A., Campana-Filho S.P. (2016). Structure, morphology and properties of genipin-crosslinked carboxymethylchitosan porous membranes. Carbohydr. Polym..

[7] Zheng Z., Zhang W., Sun W., Li X., Duan J., Cui J., Feng Z., Mansour H.M. (2013). Influence of the carboxymethyl chitosan anti-adhesion solution on the TGF-β1 in a postoperative peritoneal adhesion rat. J. Mater. Sci. Mater. Med..

[8] de Lacerda Bukzem A., Dos Santos D.M., Leite I.S., Inada N.M., Campana-Filho S.P. (2021). Tuning the properties of carboxymethylchitosan-based porous membranes for potential application as wound dressing. Int. J. Biol. Macromol..

[9] Zhuang S., Schnellmann R.G. (2005). Suramin promotes proliferation and scattering of renal epithelial cells. J. Pharmacol. Exp. Ther..

[10] Zhuang S., Lu B., Daubert R.A., Chavin K.D., Wang L., Schnellmann R.G. (2009). Suramin promotes recovery from renal ischemia/reperfusion injury in mice. Kidney Int..

[11] Korrapati M.C., Shaner B.E., Schnellmann R.G. (2012). Recovery from glycerol-induced acute kidney injury is accelerated by suramin. J. Pharmacol. Exp. Ther..

[12] Korrapati M.C., Shaner B.E., Neely B.A., Alge J.L., Arthur J.M., Schnellmann R.G. (2012). Diabetes-induced renal injury in rats is attenuated by suramin. J. Pharmacol. Exp. Ther..

[13] Dupre T.V., Doll M.A., Shah P.P., Sharp C.N., Kiefer A., Scherzer M.T., Saurabh K., Saforo D., Siow D., Casson L. (2016). Suramin protects from cisplatin-induced acute kidney injury. Am. J. Physiol.-Ren. Physiol..

[14] Korrapati M.C., Howell L.H., Shaner B.E., Megyesi J.K., Siskind L.J., Schnellmann R.G. (2013). Suramin: A potential therapy for diabetic nephropathy. PLoS ONE.

[15] He S., Rehman H., Shi Y., Krishnasamy Y., Lemasters J.J., Schnellmann R.G., Zhong Z. (2013). Suramin decreases injury and improves regeneration of ethanol-induced steatotic partial liver grafts. J. Pharmacol. Exp. Ther..

[16] Rieck P., Denis J., Peters D., Hartmann C., Pouliquen Y., Courtois Y. (1997). Fibroblast growth factor 2, heparin and suramin reduce epithelial ulcer development in experimental HSV-1 keratitis. Graefe’s Arch. Clin. Exp. Ophthalmol..

[17] Mietz H., Chévez-Barrios P., Feldman R.M., Lieberman M.W. (1998). Suramin inhibits wound healing following filtering procedures for glaucoma. Br. J. Ophthalmol..

[18] Mi F.L., Shyu S.S., Peng C.K. (2005). Characterization of ring-opening polymerization of genipin and pH-dependent cross-linking reactions between chitosan and genipin. J. Polym. Sci. Part A Polym. Chem..

[19] Yang L.-Q., Lan Y.-Q., Guo H., Cheng L.-Z., Fan J.-Z., Cai X., Zhang L.-M., Chen R.-F., Zhou H.-S. (2010). Ophthalmic drug-loaded N, O-carboxymethyl chitosan hydrogels: Synthesis, in vitro and in vivo evaluation. Acta Pharmacol. Sin..

[20] He Z., Liu C., Zhao J., Guo F., Wang Y. (2023). Enhanced gelling properties and hydration capacity of ginkgo seed proteins by genipin cross-linking. Food Chem..

[21] Del Olmo J.A., Pérez-Álvarez L., Sáez-Martínez V., Benito-Cid S., Ruiz-Rubio L., Pérez-González R., Vilas-Vilela J.L., Alonso J.M. (2022). Wound healing and antibacterial chitosan-genipin hydrogels with controlled drug delivery for synergistic anti-inflammatory activity. Int. J. Biol. Macromol..

[22] Bukhari S.M.H., Khan S., Rehanullah M., Ranjha N.M. (2015). Synthesis and Characterization of Chemically Cross-Linked Acrylic Acid/Gelatin Hydrogels: Effect of pH and Composition on Swelling and Drug Release. Int. J. Polym. Sci..

[23] Ruel-Gariépy E., Shive M., Bichara A., Berrada M., Le Garrec D., Chenite A., Leroux J.-C. (2004). A thermosensitive chitosan-based hydrogel for the local delivery of paclitaxel. Eur. J. Pharm. Biopharm..

[24] Craciun A.M., Tartau L.M., Pinteala M., Marin L. (2019). Nitrosalicyl-imine-chitosan hydrogels based drug delivery systems for long term sustained release in local therapy. J. Colloid Interface Sci..

[25] Duan J., Mansour H.M., Zhang Y., Deng X., Chen Y., Wang J., Pan Y., Zhao J. (2012). Reversion of multidrug resistance by co-encapsulation of doxorubicin and curcumin in chitosan/poly (butyl cyanoacrylate) nanoparticles. Int. J. Pharm..

[26] Patel M.P., Patel R.R., Patel J.K. (2010). Chitosan mediated targeted drug delivery system: A review. J. Pharm. Pharm. Sci..

[27] Chen J., Luo J., Feng J., Wang Y., Lv H., Zhou Y. (2024). Spatiotemporal controlled released hydrogels for multi-system regulated bone regeneration. J. Control. Release.

[28] Korsmeyer R.W. (2023). Diffusion controlled systems: Hydrogels. Polymers for Controlled Drug Delivery.

[29] Thanou M., Verhoef J., Junginger H. (2001). Oral drug absorption enhancement by chitosan and its derivatives. Adv. Drug Deliv. Rev..

[30] Chavda H., Patel C. (2010). Chitosan superporous hydrogel composite-based floating drug delivery system: A newer formulation approach. J. Pharm. Bioallied Sci..

[31] Hoare T.R., Kohane D.S. (2008). Hydrogels in drug delivery: Progress and challenges. Polymer.

[32] Peers S., Montembault A., Ladavière C. (2020). Chitosan hydrogels for sustained drug delivery. J. Control. Release.

[33] Emani S., Vangala A., Buonocore F., Yarandi N., Calabrese G. (2023). Chitosan hydrogels cross-linked with trimesic acid for the delivery of 5-fluorouracil in cancer therapy. Pharmaceutics.

[34] Kildeeva N., Chalykh A., Belokon M., Petrova T., Matveev V., Svidchenko E., Surin N., Sazhnev N. (2020). Influence of genipin crosslinking on the properties of chitosan-based films. Polymers.

[35] Harris R., Lecumberri E., Heras A. (2010). Chitosan-genipin microspheres for the controlled release of drugs: Clarithromycin, tramadol and heparin. Mar. Drugs.

[36] Khan S., Ullah A., Ullah K., Rehman N.-U. (2016). Insight into hydrogels. Des. Monomers Polym..

[37] Ibrahim Y.H.Y., Regdon G., Hamedelniel E.I., Sovány T. (2020). Review of recently used techniques and materials to improve the efficiency of orally administered proteins/peptides. DARU J. Pharm. Sci..

[38] Ways T.M.M., Lau W.M., Khutoryanskiy V.V. (2018). Chitosan and its derivatives for application in mucoadhesive drug delivery systems. Polymers.

[39] Collado-González M., González Espinosa Y., Goycoolea F.M. (2019). Interaction between chitosan and mucin: Fundamentals and applications. Biomimetics.

[40] Sander C., Nielsen H.M., Jacobsen J. (2013). Buccal delivery of metformin: TR146 cell culture model evaluating the use of bioadhesive chitosan discs for drug permeability enhancement. Int. J. Pharm..

[41] Kichou H., Bonnier F., Dancik Y., Bakar J., Michael-Jubeli R., Caritá A.C., Perse X., Soucé M., Rapetti L., Tfayli A. (2023). Strat-M^®^ positioning for skin permeation studies: A comparative study including EpiSkin^®^ RHE, and human skin. Int. J. Pharm..

[42] Mo Y.-H., Wang H., Jin S.-H., Peng K.-L., Yang Z.-M., Li P.-W., Chen Y. (2022). Preparation and properties of a fast curing carboxymethyl chitosan hydrogel for skin care. Polym. Test..

[43] Shinoda W. (2016). Permeability across lipid membranes. Biochim. Biophys. Acta-Biomembr..

[44] Hutson P.R., Tutsch K.D., Rago R., Arzoomanian R., Alberti D., Pomplun M., Church D., Marnocha R., Cheng A.L., Kehrli N. (1998). Renal clearance, tissue distribution, and CA-125 responses in a phase I trial of suramin. Clin. Cancer Res. Off. J. Am. Assoc. Cancer Res..

[45] Chen X., Yuk H., Wu J., Nabzdyk C.S., Zhao X. (2020). Instant tough bioadhesive with triggerable benign detachment. Proc. Natl. Acad. Sci. USA.

[46] Argenziano M., Haimhoffer A., Bastiancich C., Jicsinszky L., Caldera F., Trotta F., Scutera S., Alotto D., Fumagalli M., Musso T. (2019). In vitro enhanced skin permeation and retention of imiquimod loaded in β-cyclodextrin nanosponge hydrogel. Pharmaceutics.

[47] Visan A.I., Negut I. (2024). Development and applications of PLGA hydrogels for sustained delivery of therapeutic agents. Gels.

[48] Hearnden V., Sankar V., Hull K., Juras D.V., Greenberg M., Kerr A.R., Lockhart P.B., Patton L.L., Porter S., Thornhill M.H. (2012). New developments and opportunities in oral mucosal drug delivery for local and systemic disease. Adv. Drug Deliv. Rev..

[49] Xu J., Strandman S., Zhu J.X., Barralet J., Cerruti M. (2015). Genipin-crosslinked catechol-chitosan mucoadhesive hydrogels for buccal drug delivery. Biomaterials.

[50] Klecker Jr R.W., Collins J.M. (1985). Quantification of suramin by reverse-phase ion-pairing high-performance liquid chromatography. J. Liq. Chromatogr..

